# Distinct alternations of brain functional network dynamics in obsessive-compulsive disorder and schizophrenia

**DOI:** 10.1192/j.eurpsy.2021.430

**Published:** 2021-08-13

**Authors:** L. Luo, Y. Yang, Q. Gong, F. Li

**Affiliations:** 1 Huaxi Mr Research Centre (hmrrc), Department Of Radiology, West China Hospital of Sichuan University, Sichuan, China; 2 Department Of Psychiatry, West China Hospital of Sichuan University, Chengdu, China; 3 Huaxi Mr Research Centre (hmrrc), Department Of Radiology, West China Hospital of Sichuan University, Chengdu, China

**Keywords:** schizophrénia, Obsessive-Compulsive disorder, dynamic functional connectivity, Independent Component Analysis

## Abstract

**Introduction:**

Obsessive-compulsive disorder (OCD) and schizophrenia (SZ) are both severe psychiatric disorders. Though these two disorders have distinct typical symptoms, there are partial polygenic overlap and comorbidity between the two disorders. However, few studies have explored the shared and disorder-specific brain function underlying the neural pathophysiology of the two disorders, especially in the aspect of dynamics.

**Objectives:**

To explore the abnormal characteristics of the dynamic functional connectivity (dFC) in OCD and SZ as well as the association between dFC metrics and symptom severity.

**Methods:**

The resting state functional magnetic resonance imaging data of 31 patients with OCD, 49 patients with SZ, and 45 healthy controls were analyzed using independent component analysis to obtain independent components (ICs) and assigned them into eight brain networks (Figure 1), then used the sliding-window approach to generate dFC matrices. Using k-means clustering, we obtained three reoccurring dFC states (Figure 2), and state transition metrics were obtained

**Figure:**
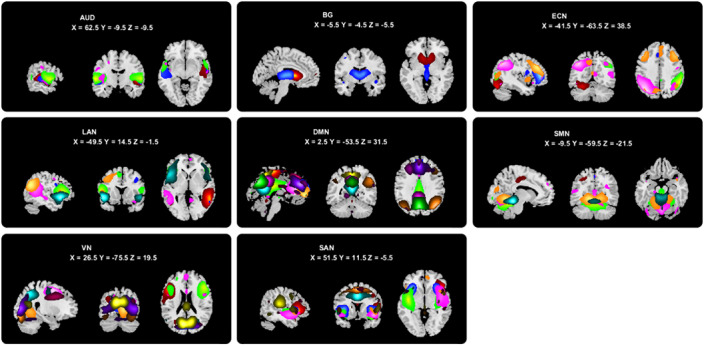

**Results:**

In a sparsely connected state (state 1), SZ showed both increased fractional time and mean dwell time than controls (P=0.047 and P=0.033) and OCD (P=0.001 and P=0.003). In a state characterized by negative FC between networks (state 2), OCD showed both increased fractional time and mean dwell time than controls (P=0.032 and P=0.013) and SZ (P=0.005 and P=0.003). Moreover, the fractional time of state 2 was positively correlated with anxiety scores in OCD (r=0.535, P=0.021, FDR corrected) (Figure 3).

**Figure:**
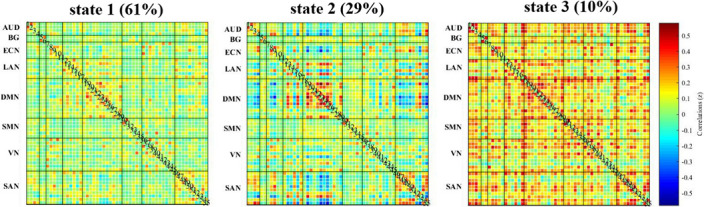

**Figure:**
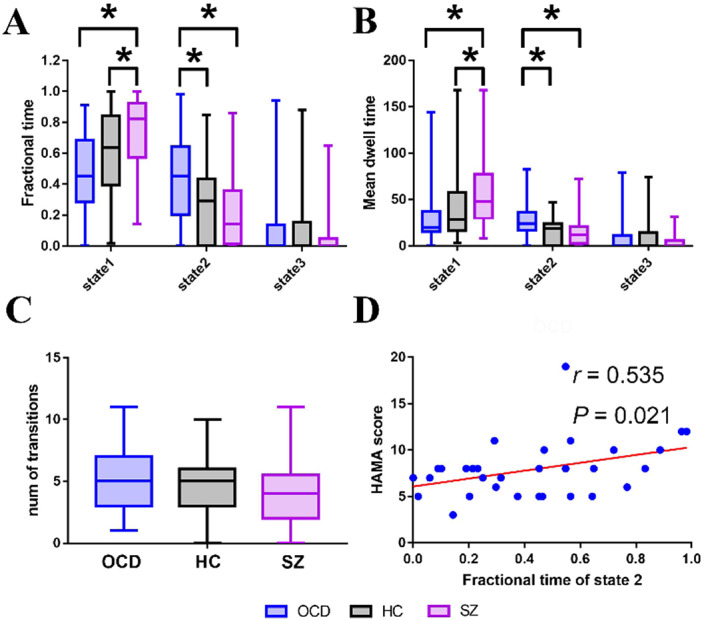

**Conclusions:**

OCD and SZ patients showed distinct alternations of brain functional dynamics.

**Disclosure:**

No significant relationships.

